# Male synthetic sling versus artificial urinary sphincter trial for men with urodynamic stress incontinence after prostate surgery (MASTER): study protocol for a randomised controlled trial

**DOI:** 10.1186/s13063-018-2501-2

**Published:** 2018-02-21

**Authors:** Lynda Constable, Nikki Cotterill, David Cooper, Cathryn Glazener, Marcus J. Drake, Mark Forrest, Chris Harding, Mary Kilonzo, Graeme MacLennan, Kirsty McCormack, Alison McDonald, Anthony Mundy, John Norrie, Robert Pickard, Craig Ramsay, Rebecca Smith, Samantha Wileman, Paul Abrams, (Chief Investigator) for the MASTER Study Group

**Affiliations:** 1 North Bristol NHS Trust, Bristol, UK; 2 University of Aberdeen, Aberdeen, UK; 3 University of Bristol, Bristol, UK; 4 Newcastle upon Tyne Hospitals NHS Foundation Trust, Newcastle, UK; 5 University College London Hospitals NHS Foundation Trust, London, UK; 6 University of Edinburgh, Edinburgh, UK

**Keywords:** Urinary incontinence, Male sling, Artificial urinary sphincter, Randomised controlled trial

## Abstract

**Background:**

Stress urinary incontinence (SUI) is a frequent adverse effect for men undergoing prostate surgery. A large proportion (around 8% after radical prostatectomy and 2% after transurethral resection of prostate (TURP)) are left with severe disabling incontinence which adversely effects their quality of life and many are reliant on containment measures such as pads (27% and 6% respectively). Surgery is currently the only option for active management of the problem.

The overwhelming majority of surgeries for persistent bothersome SUI involve artificial urinary sphincter (AUS) insertion. However, this is expensive, and necessitates manipulation of a pump to enable voiding. More recently, an alternative to AUS has been developed – a synthetic sling for men which elevates the urethra, thus treating SUI. This is thought, by some, to be less invasive, more acceptable and less expensive than AUS but clear evidence for this is lacking.

The MASTER trial aims to determine whether the male synthetic sling is non-inferior to implantation of the AUS for men who have SUI after prostate surgery (for cancer or benign disease), judged primarily on clinical effectiveness but also considering relative harms and cost-effectiveness.

**Methods/design:**

Men with urodynamic stress incontinence (USI) after prostate surgery, for whom surgery is judged appropriate, are the target population. We aim to recruit men from secondary care urological centres in the UK NHS who carry out surgery for post-prostatectomy incontinence. Outcomes will be assessed by participant-completed questionnaires and 3-day urinary bladder diaries at baseline, 6, 12 and 24 months. The 24-h urinary pad test will be used at baseline as an objective assessment of urine loss. Clinical data will be completed at the time of surgery to provide details of the operative procedures, complications and resource use in hospital. At 12 months, men will also have a clinical review to evaluate the results of surgery (including another 24-h pad test) and to identify problems or need for further treatment.

**Discussion:**

A robust examination of the comparative effectiveness of the male synthetic sling will provide high-quality evidence to determine whether or not it should be adopted widely in the NHS.

**Trial registration:**

International Standard Randomised Controlled Trial Registry: Number ISRCTN49212975. Registered on 22 July 2013. First patient randomised on 29 January 2014.

**Electronic supplementary material:**

The online version of this article (10.1186/s13063-018-2501-2) contains supplementary material, which is available to authorized users.

## Background

The male synthetic sling (male sling) is an alternative to the artificial urinary sphincter (AUS) for men with urodynamic stress incontinence after prostate surgery, but there is limited evidence of relative effectiveness and cost-effectiveness to guide choice. We aim to resolve this by directly comparing the rate of incontinence at 12 months in a non-inferiority randomised controlled trial (RCT) powered on the basis that men would accept up to 15% lesser effectiveness in return for easier device operation and possible reduced adverse effects. We will recruit patients who have decided, with their clinicians, that surgery is needed. To address feasibility of recruitment, we have devised a schedule to give early indication of our ability to recruit to target but avoiding any disruptive pause if we are successful.

### Scale of the problem in the UK and use of NHS resources

Men undergoing radical prostatectomy for prostate cancer frequently report the troublesome symptom of stress urinary incontinence (SUI). Prevalence estimates vary widely between 5% and 57% depending on definition, timing of assessment after surgery, and population characteristics. The rate of recovery of continence plateaus at around 12 months after surgery. This was confirmed in a recent large HTA-funded RCT of pelvic-floor muscle training (PFMT) in patients who suffered incontinence 6 weeks after radical prostatectomy. Subsequently, 40% had persistent UI at 1 year, with half of these (20%) having severe UI needing containment (incontinence pads or appliances) which then did not improve further during the second 12 months up to 24 months after the original surgery [1, 2].

This means that of the 6000 patients currently undergoing radical prostatectomy in the UK each year, 1200 will be using additional treatments for resultant stress incontinence beyond 12 months. UI has a major impact on quality of life, including profound loss of self-esteem together with restrictions on work, social interaction and personal relationships including sexual life. The utility value associated with a person with UI is 0.72 compared to 0.93 in a comparable age-matched population [3]. This is particularly devastating for men undergoing radical prostatectomy since they were typically without any urinary problems prior to the surgery, are fit for their age, and have a long life expectancy having generally been cured of their prostate cancer.

Unfortunately conservative treatment with one-to-one PFMT has been shown to be ineffective [1, 2], drug treatment is unproven, and men mostly cope by using containment products. Other treatments, such as injectables and inflatable balloons, have been reviewed, but there was insufficient evidence to support their use.

Surgery for persistent bothersome SUI is traditionally by artificial urinary sphincter (AUS) as the ‘gold standard’ treatment [4]. However, this is invasive, expensive and involves manipulation of a pump located in the scrotum to enable voiding. Analogous to surgery for SUI in women, synthetic slings for men have recently been developed to elevate the urethra. This is thought by some as less invasive, more acceptable to some men, and less expensive, but there is no clear evidence for its comparative effectiveness against the standard AUS. Current NHS guidance states that the male synthetic sling should only be used in RCTs against the AUS [5].

Approximately 350 men were implanted with an AUS in the UK NHS during 2010 at a cost of £9000 per procedure; £3.2 million in total. The male sling was implanted in 30 men during 2010 at a cost of £6000 per procedure; £180,000 in total.

### Evidence for surgical management for men with urinary incontinence after prostate surgery

There are no published RCTs comparing male slings with AUS. A Cochrane review found only one small, poor-quality RCT of surgery which suggested that implantation of artificial urinary sphincter (AUS) was better than an injectable bulking agent [6]. In this RCT, the men treated with AUS were more likely to be cured (18/20, 82%) than those who had the injectable treatment (11/23, 46%, OR 5.67, 95% CI 1.28 to 25.10). All other evidence comes from case series which were recently summarised by the WHO-sponsored 4th International Consultation on Incontinence [4]. This reported that the median (range) cure rate after AUS was 82% (59 to 90%, 12 series) and for male sling was 63% (13 to 86%, 20 series) [7]. A more recent review of the academic literature looked at six case series of men implanted with the AdVance® brand of male sling and reported a cure rate of 60% [8]. More recently sub-group analysis from a large case series showed that at 3 years after sling implantation, men categorised on the basis of pad usage as having ‘mild/moderate’ incontinence, had a cure/improved rate of 82% and those arbitrarily categorised as having severe incontinence had a cure/improved rate of 67% [9]. These similar cure rates, which lie within the previously reported range for all degrees of incontinence, support our intention of not using the degree of incontinence as an eligibility criterion. Results from a further recent case series suggest that the outcome of implantation of AUS is not compromised by previous insertion of a male sling [10]. As it is likely that some men in our trial, randomised to sling, may require subsequent repeat surgery, it is reassuring that their ultimate outcome is unlikely to be worse than those randomised to AUS.

We have analysed long-term follow-up data from men approached for the Men After Prostate Surgery (MAPS) trial and found that around 70% of men still reported some urine leakage 4 to 6 years after a radical prostatectomy (*N* = 579), and 39% after a transurethral resection of prostate (TURP) (*N* = 1413) (unpublished data). Of this cohort, 25% and 5% of men respectively were using pads, and 8% and 2% had leakage several times a day of a moderate or large amount of urine. A further 15 men had already had an AUS operation (of whom one required a second AUS operation), and six a male sling (of whom one required re-intervention by implantation of an AUS). In addition to these, a further 5% and 3% of men were considering surgery for incontinence.

### Evidence explaining why this research is needed now

The most recent Cochrane review showed that the efficacy of conservative treatment with PFMT was still unclear [11] and the addition of other evidence [1, 2] did not change this conclusion. As a result, a large proportion of men (around 8% after radical prostatectomy and 2% after TURP) are left with disabling incontinence which ruins their quality of life and many have no option but to continue with containment measures (27% and 6%, respectively) (unpublished data from 4-6 year follow up of MAPS responders). Surgery is, therefore, currently the only option for active management of the problem. As such, the proposed trial will provide unique robust evidence, for patients, clinicians and healthcare policy-makers, on which to base treatment and healthcare provision decisions.

The number of men undergoing radical prostatectomy for localised prostate cancer is increasing (from 2500 in 2008, to 3200 in 2010 to 5600 in 2011). This trend may continue, as localised prostate cancer case-finding using PSA testing increases, potentially leading to more men subsequently requiring surgery for prostate-cancer-treatment-related urinary incontinence. As an indication, if 50 more men required an AUS each year, this would cost the NHS an additional £450,000. While treatment with the male sling appears to be less expensive, the harms, further treatment and revision surgery needs to be taken into account to determine full comparative cost-effectiveness.

Currently, the male sling is being offered to men seeking treatment with the NHS on a haphazard basis according to surgeon enthusiasm and local arrangements. Both clinicians and patients lack the evidence required to make an informed choice between the two options and NHS policy-makers lack information on cost-effectiveness to plan service provision. The MASTER trial will fulfil the research need identified by the recent Cochrane Review [7] for adequately powered comparative RCTs of the surgical options for these men. The proposed trial will determine whether men can be confidently informed about whether implantation of the male sling gives equivalent effectiveness for cure of incontinence to the standard AUS. This will allow men and their clinicians to make an informed decision regarding the individual suitability of either option, taking into account other factors such as the relative need for subsequent re-intervention, the need to operate a control pump, and speedier recovery. As part of the trial design, we will take into account the different clinical characteristics of the men, such as type of prostate surgery, and identify factors which may influence comparative effectiveness, such as degree of incontinence. Affected men, clinicians and the NHS should benefit from the reliable evidence from the trial, to guide the choice of treatment and healthcare provision decisions, in terms of effectiveness, cost-effectiveness and adverse effects.

At present the design and function of the AUS appears optimal, as, despite attempts to improve on the existing device there are no signs of significant innovations that would have to be considered prior or during this trial. Sling technology, however, is less mature and we anticipate that during the trial recruitment period, there may be a choice of implants from different manufacturers. For that reason we will not specify which brand of sling should be used. However, it should be of the sub-urethral trans-obturator type, as currently, almost all implanted slings are of this type, and the available outcome data are chiefly for this type of sling. We feel that this research is timely since a robust examination of the comparative effectiveness of this new surgical option should provide high-quality evidence to determine whether or not it should be adopted widely in the NHS.

For a urologist to join the MASTER study, they must be uncertain regarding the best operative technique for correcting the man’s incontinence, and hence be willing to randomise the majority of patients. All the urologists must be able to perform one or both of the two operations, and be willing to randomise between them. Urologists must consider themselves competent (beyond the learning curve) and in equipoise regarding their relative merits. If surgeons only perform one procedure, they will be teamed with a surgeon who can perform the other.

### The questions which this study will address

The aim of the trial is to determine whether the male sling is non-inferior to implantation of the AUS for men who have UI after prostate surgery (for cancer or benign disease), judged primarily on clinical effectiveness but also considering relative harms and cost-effectiveness. In order to determine whether the male sling or AUS is cost-effective for the NHS in the UK, the interventions will be compared in terms of: incontinence in men after prostate surgery; the relative harms of the interventions; costs to the patients, and to the NHS, including the need for repeat surgery in both groups; and overall patient satisfaction.

#### Principal objectives

1. What is the clinical effectiveness of implantation of the male sling compared with AUS in terms of self-reported incontinence at 12 months?
2. What is the cost-effectiveness of a policy of primary implantation of the male sling compared with AUS, measured by incremental cost per quality-adjusted life-year (QALY) at 24 months?

In the long term there is a need to capture the consequences of both devices. We consider the primary outcome of the trial to be a non-inferiority comparison on rate of incontinence at 12 months. Our reason for this approach is that if the male sling is inferior (by at least the agreed margin) in the short term, then male slings will highly likely not be introduced throughout the NHS, irrespective of longer-term costs and consequences. However, if the difference in effectiveness is within the non-inferiority margin, the cost-effectiveness analysis, using outcomes over 24 months, will be required to decide on the relative worth of the interventions to the NHS.

#### Secondary objectives

3. What are the harms of each type of surgery?
4. What are the costs of the benefits and harms of each treatment policy?
5. What subsequent NHS services (including repeat surgery) are needed for men with persistent or recurrent problems?
6. What are the differential effects of the operations on other outcomes such as quality of life and general health?
7. How satisfied are the participants with each procedure?

In addition, a qualitative component has been embedded within the trial to establish patient-perceived importance of different outcomes, explore patients’ and surgeons’ perspectives on experiences of procedures and acceptable inferiority margins, and determine reasons for failure resulting in crossover to alternative surgery.

## Methods/design

### Study design

**Fig. 1:**
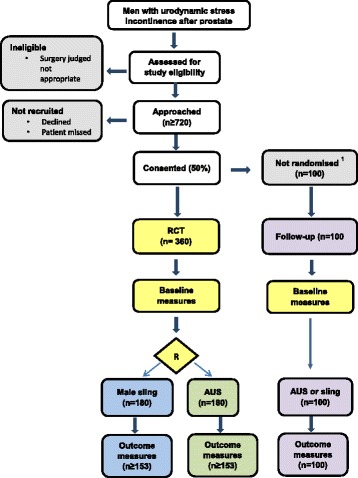
Flow diagram of study design and schedule

This trial comprises a multicentre, randomised controlled, non-inferiority trial of surgery for men with UI after prostate surgery. The trial structure is presented in Fig. 1 (flow diagram). The rationale for our proposed trial design reflects the uncertainties in the evidence base in this clinical area.

### Closure of the non-randomised cohort

The initial MASTER protocol included a non-randomised cohort (NRC) of men who did not agree to randomisation but did agree to having baseline measurements and follow-up by questionnaire. The men already recruited to the NRC have made significant contributions and will continue to be followed up. In addition, the men in the NRC have provided valuable data in the initial phase of qualitative research that will help to answer the questions listed in the qualitative research section.

### Qualitative research

A significant qualitative component is proposed for this study to underpin its development and to inform how best to interpret the results of the trial. The main aims of the qualitative components are:

1. To establish the importance of the main outcomes to patients undergoing treatment for post prostate surgery incontinence (during trial set-up at pilot sites)
2. Explore how to most appropriately evaluate non-inferiority of the procedures from the patients’ perspective using patients who were both prepared and not prepared to be randomised (before randomisation; and after refusal of randomisation. Those who refused randomisation had consented to follow-up in the non-randomised cohort while that part of MASTER was open, up to and including 27 October 2015)
3. Explore the patient experience of the two procedures
4. Explore patients’ reasons for requiring reoperation; for example, those choosing to have an AUS after a failed sling procedure
5. Explore the experience of surgeons with both procedures

Further details of the qualitative study are given in Additional file 1 (Study protocol; Appendix 2: Qualitative Study).

### Methodological research

The responses from participants and the objective findings from pad tests and urinary diaries will provide a rich data source for exploration of the correlation between patient-reported and objective outcomes, and between urinary and sexual symptoms and their effect on quality of life. This methodological research is intended to advance the controversial field of outcome measurement in lower urinary tract dysfunction, and will build upon our existing work in this area.

### Target population

Men with SUI after prostate surgery (radical prostatectomy or TURP), for whom surgery is judged appropriate, are the target population. For the purposes of the trial we will define men with mild incontinence as those not requiring surgery. There is no clear threshold for defining moderate or severe incontinence and no clear evidence of differential benefit for either intervention according to degree of incontinence. We will, therefore, include all men whose incontinence is considered, from both the patient and surgeon perspectives, to require surgery.

### Setting

Participants will be recruited from NHS secondary care urological centres throughout the UK. Discussions at a number of meetings facilitated by the relevant professional organisation, British Association of Urological Surgeons (BAUS), has gained consensus from urologists that they would be willing to randomise participants to either option. Participants will be referred by their oncological urologist, specialist cancer nurse or local urologist or continence advisor. The BAUS Section of Oncology and the Section of Female Neurological and Urodynamic Urology have been kept fully involved during the planning of this trial and have given the study their full support. However, the applicants have collaborations with colleagues throughout Europe, particularly in Belgium and The Netherlands and the protocol would be made available for their participation, if agreed, and separately funded.

### Planned interventions

Two surgical operations for male UI, the experimental technique of synthetic male sling, and the standard technique of AUS implantation will be evaluated. Divergence from pre-specified choices will be documented with reasons. All other operative variables will be described using standardised data collection forms. The surgical options have been agreed and standardised by consensus within the research team and with the recruiting urologists.

#### Male synthetic sling (male sling)

The male sling costs approximately £6000 per procedure (NHS tariff + device cost of £2000) with a typical 1-day hospital stay. It is placed under the urethra to elevate it and is held in place by passing it through the obturator foramen of the pelvic bone bilaterally. It has a passive mode of action. The aim is to stop the loss of urine on exertion and the operation is effective immediately.

#### Artificial urinary sphincter (AUS)

The artificial urinary sphincter (AUS) costs approximately £9000 per procedure (NHS tariff + device cost of £4500) with a typical 2-day hospital stay. It consists of an inflatable cuff placed around the urethra, a pressure regulating balloon to keep the cuff inflated, and a pump, placed in the scrotum, that the patient squeezes when they need to void. The aim is to close the urethra so that the patient is dry except when they wish to void. Once implanted the device is deactivated in the open position for a period of approximately 4 to 6 weeks to allow post-operative swelling to subside. The patient then returns to hospital for the device to be activated and to ensure that they are able to use the device correctly.

### Planned inclusion/exclusion criteria

#### Inclusion criteria

1. Adult men who have decided in discussion with their urologist to have surgery for urodynamic stress incontinence (USI) resulting from prostate surgery
2. Men who are willing to be randomised between male sling and AUS

#### Exclusion criteria

1. Men who have had previous male sling or AUS surgery
2. Men with unresolved bladder neck contracture or urethral stricture after prostate surgery
3. Men who do not consent to be randomised
4. Men with insufficient manual dexterity to operate AUS device
5. Men who are unable to give informed consent or complete trial documentation

### Allocation to trial groups

All eligible men referred for consideration of incontinence surgery will be identified by the consultant, dedicated research nurse, or designated team member at pre-assessment clinics, urodynamic clinics and outpatient urology clinics in each centre. The consultant/research nurse will introduce the trial to the patients and, if interest is expressed, provide details of the trial by means of the Patient Information Leaflet (PIL). Each patient will have the opportunity to discuss the trial with the local clinical team. Patients may make a decision to participate during this consultation, at a separate appointment, at a pre-admission clinic or while at home or on admission for their operation. Men who agree to be contacted at home may receive a telephone call from the local research nurse to discuss any queries. Patients who decide to participate following telephone counselling can either send their completed documents (consent and baseline questionnaire) through the post to the local team at their treating hospital or bring it with them if they are returning to hospital for another consultation or treatment. The 24-h test pads will also need to be taken into hospital for weighing at clinic visits, on the day of operation or information about the pad weight collected by telephone prior to this date.

Each man will be asked for his signed informed consent to be randomised and followed up after surgery by postal questionnaires and clinical review. The PIL and the consent form will both refer to the possibility of long-term follow-up and being contacted about other research if the man is willing, e.g. data collection from hospital and NHS records, including Office of National Statistics (ONS) and NHS central registers.

All participants who enter the trial will be logged with the central Study Office and given a unique Study Number. Randomisation will utilise the existing proven remote automated computer randomisation application at the study administrative centre in the Centre for Healthcare Randomised Trials (CHaRT, a fully registered UK CRN clinical trials unit) in the Health Services Research Unit, University of Aberdeen. This randomisation application will be available both as a telephone-based Interactive Voice Response (IVR) system and as an Internet-based service.

Randomisation will be computer-allocated and minimised on:

- Type of prostate surgery (radical prostatectomy or TURP)
- Whether or not they have had radiotherapy in addition to surgery
- Centre

All participants who consent to enter the trial will complete baseline questionnaires, including measurement of urinary and sexual symptoms, a urinary bladder diary, and have an objective 24-h pad test carried out prior to randomisation. Participants who consent to randomisation will then be randomised to receive a male sling or AUS.

### Methods to protect against sources of bias

#### Randomisation (avoiding selection bias)

Participants will be logged and registered on the trial database prior to trial entry. Randomisation will be by secure remote third-party either via computer allocation using a web application or telephone. Randomisation will be carried out as close to the time of surgery as is practical, taking into account the standard procedures in each centre for ordering implants and arranging theatre schedules.

#### Ensuring standardisation of intervention and outcome measurement (performance bias)

##### Investigators

The basic acceptance criteria for participating urologists is that they must be uncertain regarding the best operative technique for the majority of patients, and they must be competent to perform the operations to be compared (i.e. beyond the learning curve), as judged by themselves and/or trial-appointed trainers. The investigators are specialist urologists who will be responsible for recruiting and randomising men. All will be experienced in performing both operations, or if a surgeon performs only male sling or AUS, then they will be ‘paired’ with a local urologist who performs the other procedure, thereby guaranteeing that all participants will be operated on by the surgeon experienced in the operative technique to which he is randomised.

##### Standardisation of surgical techniques

The surgical procedures and other operative variables have been standardised as much as possible by using agreed protocols developed by the urological grant holders (Professors Abrams, Drake, Mundy and Pickard). Any deviations from agreed protocols will be recorded. All investigators are experienced urological surgeons. Investigators, who are experienced in male sling surgery, will deliver any additional training if required. The clinical grant applicants will ensure standardisation of existing techniques and outcome measures, including the use of new devices.

The research nurses and/or the surgeons will complete a theatre Case Report Form (CRF) at the time of surgery, to ensure a complete record of all surgical techniques and materials used, and any intra-operative difficulties or complications. The research nurses in each centre will ensure completeness and accuracy of data entry using remote data capture via a trial web-based portal at the Study Office in Aberdeen, authored and managed by the Centre for Healthcare Randomised Trials (CHaRT), the UK CRN-registered trials unit in Aberdeen.

As this is a pragmatic trial, post-operative care will be according to local centre practice, but clinical and resource-use data will be recorded.

#### Loss to follow-up (attrition bias)

Loss to follow-up in our previous trial of conservative treatment for men with UI after prostate surgery [1, 2] was 5 to 10% at 1 year. However, a less optimistic estimate of 15% loss to follow-up has been used in the sample size calculations. We will take very active measures to minimise such loss, such as phoning the participants, using retention incentives and checks with their general practitioners (GPs). In addition, we will obtain consent from the participants to enable us to access centrally held NHS data; for example, via the NHS Strategic Tracing Service in England and Wales, and using Community Health Index (CHI) numbers from the Information Services Division in Scotland.

#### Other sources of bias (detection bias)

After randomisation, participants will not be told of their allocation before surgery unless they specifically request this information. Blinding in theatre is not possible given that this is a surgical procedure trial with different implantation devices. After surgery, group allocation cannot be concealed from the participant or the ward staff due to the nature of the device. Outcome assessment is largely by participant self-completed questionnaire, so avoiding interviewer bias.

Research staff will be blinded to allocation while conducting data collection for outcomes (e.g. pad test weighing), performing data entry and analysis, and by using Study Numbers only to identify participants, questionnaires, diaries and pads. Participants will be asked not to reveal information about their surgical treatment. Staff will be asked to record whether or not they knew which operation was performed before undertaking outcome assessments. All participants will be actively followed up, with analysis based on the intention-to-treat principle. All analyses will be clearly predefined to avoid bias.

### Sample size

There is a lack of robust evidence from comparative studies on which to base the trial sample size calculation. For the primary outcome (incontinence), limited evidence from case series suggests that 20% of men would still be incontinent 12 months after AUS. For male slings, after primary implantation the percentage of men who are incontinent is thought to be 35%.

For our chosen non-inferiority comparison at 12 months, a trial with 310 participants will allow us to be 90% sure that the lower limit of a two-sided 95% confidence interval will exclude the possibility that the AUS is more effective by a margin of 15% or more. Allowing for 15% loss to follow-up after enrolment we plan to recruit 180 participants per group into the trial. This sample size will allow the detection of a difference equivalent to 0.25 of a standard deviation (SD) at 80% power between the groups for the key secondary outcome, International Consultation of Incontinence Questionnaire (ICIQ) at 24 months.

## Subsequent arrangements

### Informing key people

Following formal trial entry:

The Study Office will:

1. Inform the participant’s GP (by letter) enclosing information about MASTER and the Study Office contact details

The local research nurse/recruitment officer and/or urologist will:

1. File the hospital copy of the consent form in the hospital notes along with information about MASTER
2. Inform the ward and theatre staff as appropriate of the participant’s entry to the trial and details of the intervention allocation (theatre only)
3. Use the MASTER Internet database to enter data regarding the participant, including data required to complete randomisation; and intra-operative and post-operative information abstracted from local medical records
4. Maintain and archive study documentation at the site. A copy of the signed consent form is returned to the Study Office in Aberdeen after database entry

### Monitoring the participants

Participants will be contacted by telephone, post or email as appropriate. In case of non-return of questionnaires, or non-attendance at outpatient appointments, attempts will be made by staff at the Study Office to trace the participant directly using these means or indirectly by contacting the GP.

### Notification by GPs

GPs are asked to contact the Study Office if the participant moves, becomes too ill to continue or dies, or any other notifiable or adverse event occurs. Alternatively, staff at the Study Office may contact the GP.

### Offices for National Statistics (HES (Hospital Episode Statistics) data in England, ISD (Information Statistics Division) data in Scotland)

Consent will be sought from all participants to trace their medical records and addresses from local records and centrally held computerised databases. This should facilitate long-term follow-up.

### Ethical arrangements

We believe that the proposed research does not pose any specific risks to individual participants nor does it raise any extraordinary ethical issues.

## Data collection and processing

Follow-up will continue for 24 months from the date of randomisation including those who agreed to enter the non-randomised cohort whist that part of MASTER was open (up to and including 27 October 2015). It is not part of this protocol or the current study to follow up the men beyond this time. However, consent will be sought to make this possible in the future, and long-term follow-up is planned.

### Proposed outcome measures

The outcomes are similar to those piloted and used successfully in MAPS, with the addition of relevant surgical outcome measures. The primary outcome uses the ICIQ-UI Short Form (SF).

#### Primary outcome measures

The primary clinical outcome is participants’ report of UI at 12 months measured by a response indicating any loss of urine to either of the two questions: ‘How often do you leak urine?’ and ‘How much urine do you leak?’ in the validated ICIQ-SF [12].

The primary economic outcome measure of cost-effectiveness is incremental cost per QALY at 24 months based on responses to the EuroQol Group’s five-dimension health status questionnaire (EQ-5D™) [13].

#### Secondary outcome measures

##### General

- Immediate and late post-operative morbidity; blood loss
- Complications related to devices. e.g. urethral erosion or infection
- Other adverse effects or complications
- Operating time
- Length of hospital stay
- Number of readmissions to hospital
- Time until resumption of usual activities
- Need for further surgery for urinary incontinence
- Time to further surgery
- Satisfaction with treatment (ICIQ-satisfaction)

##### Urinary outcomes

- Urinary incontinence (ICIQ-UI SF Score and types of incontinence)
- Use of pads
- 24-h pad test (weight of urine lost)
- Lower urinary tract symptoms (frequency, nocturia, urgency, and voiding symptoms such as slow stream and hesitancy (ICIQ-Male Lower Urinary Tract Symptoms (MLUTS)).

##### Sexual function outcomes

- Sexual function (ICIQ-MLUTSsex)

##### Quality-of-life outcome measures

- Condition-specific quality-of-life measures (incontinence from ICIQ-UI SF, and sexual from ICIQ-MLUTSsex)
- General health measures (SF12 and EQ-5D)™

##### Economic outcome measures

- Need for alternative management for incontinence or voiding dysfunction (e.g. PFMT; further surgery; use of pads, drugs, or sheath, indwelling or intermittent catheters)
- Cost and use of NHS services
- Cost to the participants and their families/carers
- QALYs estimated from the responses to the EQ-5D™
- The incremental costs, QALYs and incremental cost per QALY derived by the economic model over a longer-term time horizon

In addition, all participants who have surgery (including non-randomised men who entered the NRC while it was open) will be asked to consent to long-term follow-up, including the use of computerised NHS records and other routine data sources.

#### Measurement of outcomes (Fig. 2)

Outcomes will be assessed by participant-completed questionnaires and 3-day urinary bladder diaries at baseline, 6, 12 and 24 months. The 24-h urinary pad test will be used at baseline as an objective assessment of urine loss, measured by pad weighing in grams per 24 h. The research nurse and/or urologist will complete a CRF at the time of surgery providing details of the operative procedures, complications and resource use in hospital. At 12 months the randomised men will also have a review appointment with their urologist and/or research nurse to evaluate the results of surgery (including another 24-h pad test)*,* and to identify problems or the need for other treatment. This may occur via the telephone if participants have already been discharged by the local centre before this date or pads weighed at a non-research centre closer to their home (provided the equipment used to do this is sufficiently accurate).

**Fig. 2:**
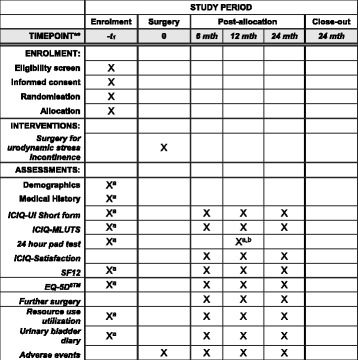
Standard Protocol Items: Recommendations for Interventional Trials (SPIRIT) Figure: trial timeline for recruitment, intervention, assessment and follow-up

Economic outcomes will be assessed using standard economic methods plus trial-specific data collection described earlier. We are using standardised outcome instruments developed by the International Consultation on Incontinence (ICI) for urinary and sexual symptoms [12]. The components and timing of follow-up measures are shown in Fig. 2 (Standard Protocol Items: Recommendations for Interventional Trials (SPIRIT) Figure [14]).

### Questionnaires and Case Report Forms (CRFs)

#### Questionnaires for participants

Participants will be asked to complete a baseline questionnaire and diary before surgery. Content will include:

1. Healthcare utilisation questions (including GP consultations and hospital visits/admissions, use of other services)
2. Personal costs (pad use, catheter use, over-the-counter medication, other healthcare services)
3. EQ-5D™
4. Urinary symptoms (ICIQ-MLUTS, urinary leakage ICIQ-UISF, and effect on quality of life, ICIQ-qol, http://www.iciq.net/structure.html)
5. Sexual symptoms (ICIQ-MLUTSsex); http://www.iciq.net/ICIQ.MLUTS.html

The follow-up questionnaires and diaries at 6, 12 and 24 months will repeat the baseline questions and, in addition, will enquire about:

1. Complications and adverse effects
2. Need for further treatment for incontinence or complications, including further surgery

The follow-up questionnaire at 12 and 24 months will repeat the questions and in addition will enquire about:

1. Satisfaction with surgery results and willingness to recommend to a friend

#### Urinary diaries

Participants will be asked to complete urinary diaries at each questionnaire time point, including frequency of micturition, leakage and nocturia, use of pads and wetting of clothes.

### Case Report Forms (CRFs)

#### Baseline CRF

At baseline, the urologist and/or research nurse will complete a CRF with the following content:

##### Pre-operative

1. Contact details, GP address, telephone numbers
2. Urological and surgical history
3. Urodynamics
4. Pad tests

##### Intra-operative

1. Intra-operative data including date of admission and operation
2. Operative procedures and theatre time
3. Catheter use
4. Complications

##### Post-operative

1. Pain relief, infection, haematoma, other complications
2. Date of discharge

#### 12-month clinical review assessment form

At 12 months after surgery, all men will be reviewed by the urologist and/or the research nurse:

1. Clinical findings (pad tests)
2. Complications and adverse events

#### Serious adverse event (SAE) Case Report Form

Serious adverse events will be coded and recorded using a standard SAE CRF at the behest of a local urologist. The SAE form will be used to record details of any serious adverse events related to the incontinence surgery/procedure undertaken as part of MASTER.

### HES and ISD data

After the last man has been recruited, we will run periodic checks for operations, diagnoses and hospital admissions with centrally collected data, to supplement and validate data collected from the participants, and to set up mechanisms for long-term follow-up.

### Data processing

Research nurses will enter locally collected data in the centres. Staff in the Study Office will work closely with local research nurses to ensure that the data are as complete and accurate as possible. Follow-up questionnaires to men will be sent from, and returned to, the Study Office in Aberdeen. Extensive range and consistency checks will further enhance the quality of the data.

### Withdrawal procedures

Participants may withdraw from any aspect of the trial.

## Analysis plans

### Statistical analysis

All analyses will be based on the intention-to-treat principle, analysing participants in the groups to which they were randomised. All missing data will be imputed at baseline using appropriate imputation methods. Missing items on the health-related outcome measures will be treated as per the instructions for that particular measure but without imputation for other missing follow-up data. All outcomes will be described with the appropriate descriptive statistics where relevant: mean and standard deviation for continuous and count outcomes, or medians and inter-quartile range if required for skewed data; numbers and percentages for dichotomous and categorical outcomes (for example, subjective recurrence of incontinence).

Analysis of the primary outcome (number of participants with UI) will estimate the mean differences at 12 months after surgery (and 95% confidence intervals) between the two intervention groups using a general linear model that adjusts for the minimisation covariates and other important prognostic covariates, including the baseline symptom score, at 12 months after surgery. A two-sided statistical significance (2*P* < 0.05) will be sought. A similar analysis will be used to analyse the data at 6 and 24 months.

All secondary outcomes will be analysed in a similar manner but using the appropriate generalised linear model (for example, logistic regression for dichotomous data such as subjective failure, Poisson or negative binomial regression for count data such as number of nights in hospital) or time to event methods (e.g. Cox regression on time to further surgery) where required. We will explore analysing outcomes at all time points simultaneously using, for example, Generalised Estimating Equations or Generalised Linear Latent and Mixed Models, and relevant link functions to explore changes in outcome over time. Further details about the statistical analysis will be outlined in the Statistical Analysis Plan.

#### Planned subgroup analyses

Subgroup analysis according to type of prostate surgery will be considered within the following groups:

- Radical prostatectomy or TURP
- Amount of urine leaked per 24 h at baseline, above, and below or equal to 250 g per 24 h

Heterogeneity of treatment effects amongst subgroups will be tested for using the appropriate subgroup by treatment group interactions [15]. Stricter levels of statistical significance (2*P* < 0.01) will be sought, reflecting the exploratory nature of these analyses.

All study analyses will be according to a Statistical Analysis Plan that will be agreed in advance by the Trial Steering Committee (TSC) and the Data Monitoring Committee (DMC).

#### Proposed frequency of analyses

A single main analysis will be performed at the end of the trial when all 24-month follow-up has been completed. An independent DMC will review confidential interim analyses of accumulating data at its discretion but at least annually. A major consideration for the DMC will be to monitor the 12-month primary outcome (i.e. the non-inferiority margin).

### Economic evaluation

The trial will include a formal economic evaluation assessing the costs and cost-effectiveness of the interventions compared from the perspectives of the NHS and the participants and their families. Resource-use data collected will include the cost of the intervention and the use of primary and secondary NHS services by the participants, including further referral for subsequent additional specialist management. Health service costs refer to those incurred directly by the NHS due to the surgery and subsequent appointments and procedures. Personal costs to the participants (such as costs of travelling to appointments and work/social restrictions) will also be investigated.

Resource use will be recorded prospectively for every participant within the study. For the surgical interventions, operative details will be recorded at the time of surgery (e.g. time the surgery takes, the time spent in recovery, grade of surgeon and assistant, grade of anaesthetist). A parallel exercise will establish resources used immediately before, during and after (i.e. in recovery) the operation, e.g. other staff, consumables (surgical requisites) and capital (costs associated with using the theatre facilities, costs of using reusable equipment). Costs to the participants will be collected using a questionnaire based on one developed by the UK working party on patient costs. The use of secondary care services (e.g. length of hospital stay, outpatient appointments, and readmission) will be abstracted from patient notes or questionnaires. The use of primary care services, including medications prescribed will be collected using a patient questionnaire. Unit costs/prices will be obtained using published estimates for healthcare services and/or interventions.

A generic instrument (the EQ-5D™) will be used to measure health state. Trial participants will be asked to complete the EQ-5D™ at baseline and at 6, 12 and 24 months after their operation. This instrument will provide the quality of life weights to compute the QALYs.

Incremental cost-effectiveness ratios will be computed comparing the cost of the interventions. The difference in effectiveness will be expressed in terms of the number of participants who are still incontinent at 24 months. These data will be based on responses to either of two questions relating to the loss of urine, retrieved from the participant questionnaires. Incremental cost-utility ratios will be computed comparing the interventions. The difference in utility will be expressed in terms of QALYs at 24 months. Where appropriate, the analysis of incremental costs, effectiveness and cost-effectiveness will be based on similar statistical models as those outlined in the Statistical Analysis Plan above. This ‘within’ trial analysis will include both deterministic and stochastic sensitivity analyses to explore statistical and other forms (e.g. around unit costs or the source of utility estimates) of uncertainty.

An economic model which considers a longer time horizon will also be developed to provide additional information for policy-makers. In the model, the findings of the trial will be extrapolated to the participant’s life time. The model will describe care pathways that men may follow and will include the initial surgery and any subsequent treatments. The structure of the model will be developed in collaboration with the expert panel of service users, patients, clinicians and trial collaborators. Parameter estimates for relative effectiveness up to two years, costs and utilities will be derived from the trial data. Data from the trial will be supplemented with data from other sources (e.g. Cochrane review, other future RCTs). These data will be assembled systematically and will follow guidelines for good practice [16].

Outcomes in the model will be expressed in terms of an incremental cost per QALY. Parameter uncertainty will be integrated by the incorporation of probability distributions into the model and involve Monte Carlo simulation. Other forms of uncertainty, such as that associated with choices made about the structure of the model, discount rate, etc. will be addressed through sensitivity analysis. The base case and sensitivity analyses will be presented as cost-effectiveness acceptability curves. The model will also be used to identify priorities for further research by investigating the expected value of information.

## Recruitment rates and milestones

**Fig. 3:**
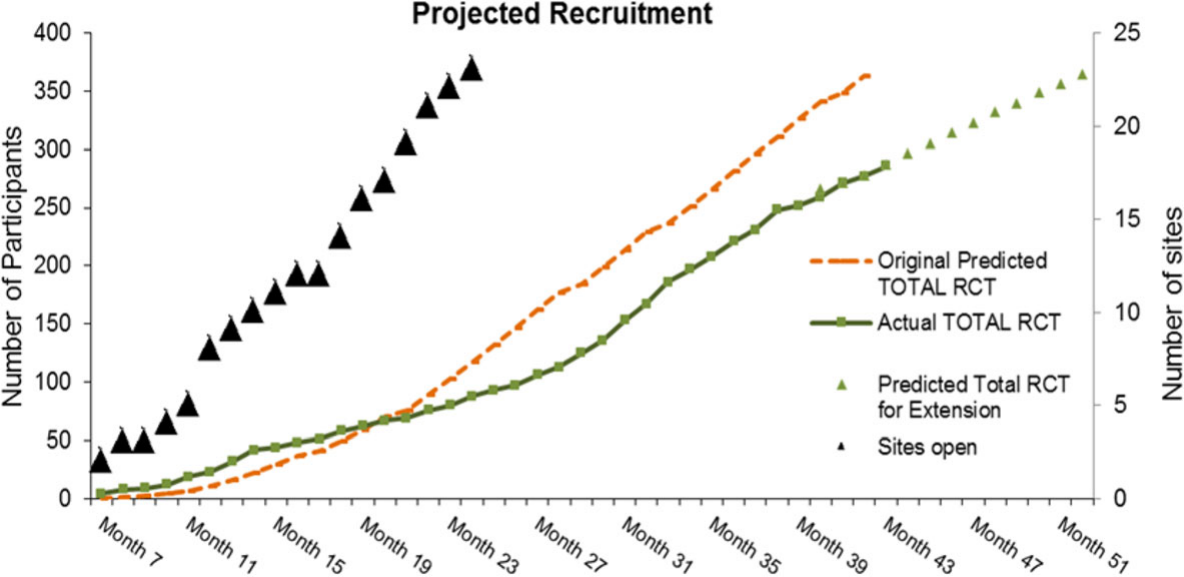
Site accrual and recruitment projections

Figure 3 shows the projected recruitment of centres and participants, and the projected number of men to be approached. Five centres will be established relatively early in the project as an internal pilot followed by roll out to the others over the subsequent months.

An internal pilot is included, primarily designed to verify that recruitment is possible. We will make a decision about feasibility at around month 15 when 82 centre months have been accrued and approximately 43 participants randomised. We anticipate that this should include ‘steady state’ data from the five selected pilot centres and initial data from up to 10 other centres more recently set up. This rule will try to statistically assess the accumulating recruitment to see if it is consistent with the required rate to recruit on time and budget to the full trial. It would take the form of ‘If recruitment is at least 37 of the anticipated 43, continue unchanged to full study; if between 26 and 36, then consider modifying the design; if 25 or less, consider that the trial is not feasible’. If the trial progressed as planned we would anticipate having 117 randomised participants by month 24, 281 patients by month 36 and the remaining 79 patients by month 42, making a total of 360 participants.

### Extension to recruitment

Due to the slower than anticipated recruitment, a 9-month extension has been approved. Based on a conservative estimate of the recruitment trend, nine randomised participants per month, for an additional 9 months will resulted in achieving target recruitment (Fig. 3).

**Fig. 4:**
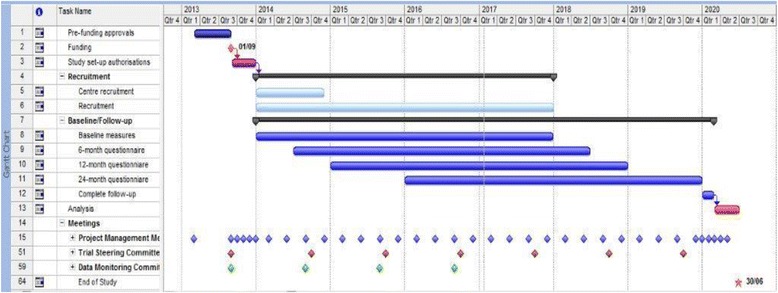
Gantt chart

The funding for the trial started on 1 September 2013 and the duration is 82 months. The Gantt Chart (Fig. 4) illustrates the main milestones: pre-funding: multicentre research ethics and central Research and Development (R&D) approvals; months 1–6: set-up office, assemble team, and establish the first five centres; months 7–16: aim to establish the trial in all centres; months 7–51: identify and recruit 360 participants; months 13–75: follow-up at 6, 12 and 24 months after surgery; months 76–87: final reminders; months 76–82: complete data collection, analysis and dissemination.

The trial will continue to 31 May 2020 with the possibility of longer-term follow-up through a separate funding application.

## Organisation

A detailed plan and timetable of study organisation is given in the Gantt chart (Fig. 4).

The Gantt chart indicates when it is anticipated that the major study events will occur, including recruitment, trial progress and meetings. There will be approximately 3-monthly project management group meetings, six meetings of the Steering Committee and four of the Data Monitoring Committee. Two meetings are planned for collaborators (including urologists, local research nurses and consumer participants); the first, timed to occur when all the sites have been identified, and the second when results are available. There will also be a training meeting for the recruitment nurses.

These time-related milestones will be used to enable close monitoring of progress.

### Local organisation in centres

#### Lead urologist (local principal investigator)

Each collaborating centre will identify a lead urologist who will be the point of contact for that centre.

The responsibilities of this person will be to:

- Establish the study locally (for example, by getting agreement from clinical colleagues; facilitate local regulatory approvals; identify, appoint and train a local research nurse; and inform all relevant local staff about the study (e.g. other consultant urologists, junior medical staff, secretaries, ward staff)
- Take responsibility for clinical aspects of the study locally (for example, if any particular concerns occur)
- Identify patients who are eligible to participate in the trial, explain the different surgery options to them, and ensure that study documentation has been provided and that informed consent has been obtained
- Notify the Study Office of any unexpected, serious clinical events which might be related to trial participation and assess the implications of events leading to these for the safety of other trial participants
- Provide support, training and supervision for the local research nurse(s)
- Represent the centre at the collaborators’ meetings

#### Local research nurse

Each collaborating centre will appoint a local research nurse to organise the day-to-day recruitment of participants to the trial.

The responsibilities of this person will be to:

- Keep regular contact with the local lead urologist, with notification of any problem or unexpected development
- Maintain regular contact with the MASTER Study Office
- Keep local staff informed of progress in the trial
- Contact potential participants by: providing the PIL to patients being admitted electively for UI surgery; identifying any eligible patients at pre-assessment clinics or on the ward while they are in hospital for their surgery; explain the study and the potential for participation in a trial if they are eligible; explaining what is intended by research access to their NHS data; and describing the possibility of long-term follow-up and participation in other research
- Obtain the patient’s written consent
- Keep a log of whether eligible men are recruited or not (with reasons for non-participation)
- Collect baseline data describing the participants, log this information in the web-based MASTER database and send paper copies to the Study Office along with the original signed consent forms in a timely manner
- Use this information to randomise the participants using the web-based MASTER database or telephone
- Ensure operative and post-operative data (including 12-month clinic CRF hospital readmission form,, withdrawal form and SAE form) are collected and recorded in the web-based MASTER database, and send paper copies to the Study Office in a timely manner
- File relevant study documentation (e.g. consent forms) in the participant’s medical records
- Organise and supervise alternative recruiters in case of holiday or absence
- Represent the centre at the collaborators’ meetings

### Study co-ordination in Aberdeen

#### The Study Office Team

The Study Office is in CHaRT, Health Services Research Unit in Aberdeen and provides day to day support for the clinical centres. It is responsible for all data collection (such as mailing questionnaires), follow-up, data processing and analysis. It is also responsible for providing and maintaining the randomisation service, and communicating with the sites about MASTER-specific issues. We will produce a yearly MASTER Newsletter for participants and, in addition, regular meetings with research nurses and local collaborators to inform everyone of progress and maintain enthusiasm.

The MASTER Study Office Team (Aberdeen-based grant holders and study office members, plus the chief investigator (CI)) will meet formally approximately monthly during the course of the study to ensure smooth running and trouble-shooting.

#### The Project Management Group (PMG)

The study is supervised by its Project Management Group. This consists of the grant holders and representatives from the Study Office. Observers may be invited to attend at the discretion of the Project Management Group. They plan to meet or hold a teleconference every 3 months on average.

#### The Trial Steering Committee (TSC)

The study is overseen by an independent Trial Steering Committee (TSC). The membership comprises the four independent members (including the Chairman), and the CI (or a deputy). The other grant holders, a representative from the sponsoring institution and the funders (the HTA) may also attend, as may other members of the MASTER Study Office or members of other professional bodies at the invitation of the Chair.

### Research governance, data protection and sponsorship

#### Research governance

The trial will be run under the auspices of CHaRT based at the Health Services Research Unit, University of Aberdeen. This will ensure compliance with Research Governance, and provide centralised trial administration, database support and economic and statistical analyses. CHaRT is a registered Clinical Trials Unit with particular expertise in running multicentre RCTs of complex and surgical interventions.

The CI will ensure, through the TSC, that adequate systems are in place for monitoring the quality of the study (compliance with good clinical practise (GCP)) and appropriate expedited and routine reports of adverse effects, to a level appropriate to the risk assessment of the study.

#### Data protection

The trial will comply with the Data Protection Act 1998 and regular checks and monitoring are in place to ensure compliance. Data are stored securely in accordance with the Act and archived to a secure data-storage facility. The consent form will state that other researchers may wish to access (anonymised) data in the future. The senior IT manager (in collaboration with the CI) will manage access rights to the data set. Prospective new users must demonstrate compliance with legal, data protection and ethical guidelines before any data are released. It is anticipated that anonymised trial data will be shared with other researchers to enable international prospective meta-analyses.

#### Sponsorship

The study is sponsored by the North Bristol NHS Trust.

#### Retention of data

It is intended to follow-up the whole cohort of participants for at least 10 years, and data will be retained as long as necessary for this purpose. Permissions will be sought from the relevant research governance bodies and the Ethics Committee.

### Data and safety monitoring

#### Data Monitoring Committee

There is a separate and independent Data Monitoring Committee (DMC). It is anticipated the members will meet once to agree terms of reference and on at least three further occasions to monitor accumulating data and oversee safety issues. This committee is independent of the study organisers and the TSC. During the period of recruitment to the study, interim analyses will be supplied, in strict confidence, to the DMC, together with any other analyses that the committee may request. This may include analyses of data from other comparable trials. In the light of these interim analyses, the DMC will advise the Steering Committee if, in its view:

1. One of the methods of surgery has been proved, beyond reasonable doubt,^1^ to be different from the control (standard management) for all or some types of participants (in respect of either effectiveness or unacceptable safety concerns), and
2. The evidence on the economic outcomes is sufficient to guide a decision from healthcare providers regarding recommendation of which operation to choose

The TSC can then decide whether or not to modify intake to the trial. Unless this happens, however, the TSC, PMG, clinical collaborators and Study Office staff (except those who supply the confidential analyses) will remain ignorant of the interim results.

The frequency of interim analyses will depend on the judgement of the Chairman of the DMC. However, we anticipate that there might be two interim analyses and one final analysis.

The Chairman and the other independent members are appointed after confirmation by the HTA.

#### Safety concerns

The MASTER trial involves surgical operations for UI which are established in clinical practice. Adverse effects may occur after any type of surgery. The relevant guidelines for reporting serious adverse events will be followed.

Collaborators and participants may contact the Chairman of the TSC through the Study Office about any concerns they may have about the study. If concerns arise about procedures, participants or clinical or research staff (including risks to staff) these will be relayed to the Chairman of the DMC.

#### Safety definitions

An adverse event (AE) is defined as any untoward medical occurrence in a participant, not necessarily having a causal relationship.

Adverse events are not:

- Continuous and persistent disease or symptom, present before the trial, which fails to progress
- Signs or symptoms of the disease being studied (in this case incontinence), or
- Treatment failure

An adverse event is defined as ‘serious’ (SAE) if it:

- Results in death
- Is life threatening
- Requires or prolongs inpatient hospitalisation
- Results in persistent/significant disability/incapacity
- Is otherwise considered medically significant by the investigator

Within MASTER, an adverse event is defined as ‘related’ if it occurs as a result of a procedure required by the protocol, whether or not this procedure is the specific intervention under investigation and whether or not it would have been administered outside the study as normal care.

##### Expected adverse events

In this study the following adverse events are potentially expected:

Possible (expected) adverse events during or associated with surgery include:

- Excess blood loss (> 500 ml) or transfusion
- Injury to organs (e.g. bladder, bowel, urethra), blood vessels or nerves
- Anaesthetic complications
- Death

Possible (expected) adverse events following surgery include:

- Excess blood loss (> 500 ml)
- Blood transfusion
- Haematoma formation
- Prolongation of post-operative catheterisation
- Recatheterisation
- Urinary retention/voiding difficulties requiring surgical intervention; urinary retention/voiding difficulties not requiring catheterisation or surgery
- Bowel obstruction
- Constipation
- Thrombosis/deep vein thrombosis (DVT)/pulmonary embolism
- Urinary tract infection
- Wound infection
- Wound breakdown
- Other infection (sepsis, septicaemia, abscess)
- New, bothersome urinary tract symptoms
- Division of male sling
- Device exposure/extrusion which requires no treatment or conservative treatment
- Device exposure/extrusion requiring hospitalisation for surgical removal of the device
- Abnormal pain (acute or chronic, e.g. buttock or groin pain/sciatica)
- New, bothersome sexual problems
- Death

#### Recording and reporting of SAEs in MASTER

##### Recording

Non-serious events will be recorded in the CRFs and participant questionnaires and collated for the sponsor, but these will not be followed up further. Planned primary care or hospital visits for conditions other than those associated with UI or consequence of surgery will not be collected or reported. Additional hospital admissions (planned or unplanned) associated with further UI treatment (e.g. further surgery) will be recorded as an outcome measure, but will not be reported as serious adverse events. Relevant data will be collected on the additional hospital admissions CRF.

Any SAEs related to the participants’ UI surgery that are not further interventions (e.g. if a participant is admitted to hospital for treatment of infection) will be recorded on the serious adverse event form. In addition, all deaths for any cause (related or otherwise) and related life-threatening events will be recorded on the serious adverse event form.

##### Reporting responsibilities of the CI

When the SAE form is uploaded onto the trial website, the CI or trial manager will be automatically notified. If, in the opinion of the local principal investigator (PI) and the CI, the event is confirmed as being serious and related and unexpected, the CI or trial manager will notify the sponsor within 24 h of receiving the SAE notification. The CI or trial manager will notify the sponsor of expected SAEs in a timely fashion. The sponsor will provide an assessment of the SAE. The CI (or trial manager) will report any related and unexpected SAEs to the main Research Ethics Committee (REC) and the DMC within 15 days of the CI becoming aware of it. All related SAEs will be summarised and reported to the Ethics Committee, the Funder and the Trial Steering Committee in their regular progress reports.

## Discussion

The MASTER trial is the largest RCT evaluating the effectiveness of AUS and the male sling for men who have SUI after prostate surgery (for cancer or benign disease). The results of MASTER will be used to determine optimal treatment decisions for these men.

## Trial status

The first participant was randomised into the trial on 29 January 2014, and the trial is currently open to recruitment in 28 UK centres, with the last participant follow-up expected in 31 December 2019.

MASTER protocol Version 4: 15 June 2017.

## Additional files

Additional file 1: Male synthetic sling versus artificial urinary sphincter trial. (PDF 540 kb)

Additional file 2: SPIRIT 2013 Checklist: recommended items to address in a clinical trial protocol and related documents. (PDF 57 kb)

## Abbreviations

AE: Adverse event
AUS: Artificial urinary sphincter
BAUS: British Association of Urological Surgeons
CHaRT: Centre for Healthcare Randomised Trials
CHI: Community Health Index
CI: Chief investigator
CRF: Case Report Form
DMC: Data Monitoring Committee
EQ-5D^TM^: EuroQol Group’s five-dimension health status questionnaire
GCP: Good Clinical Practice
GP: General Practitioner
HES: Hospital Episode Statistics
HTA: Health Technology Assessment
ICI: International Consultation on Incontinence
ISD: Information Statistics Division
ISRCTN: International Standard Randomised Controlled Trial Number
IVR: Interactive Voice Response (randomisation)
MAPS: Men After Prostate Surgery
MLUTS: Male Lower Urinary Tract Symptoms
NETSCC: NIHR Evaluation, Trials and Studies Coordinating Centre
NHS: National Health Service
NIHR: National Institute for Health Research
NRES: National Research Ethics Service
ONS: Office of National Statistics
OR: Odds ratio
PFMT: Pelvic-floor muscle training
PI: Principal investigator
PMG: Project Management Group
QALY: Quality-adjusted life year
R&D: Research and Development
RCT: Randomised controlled trial
REC: Research Ethics Committee
REC: Research Ethics Committee
SAE: Serious adverse event
SD: Standard deviation
SF: Short Form
SIS: Surgical Information Sheet
TSC: Trial Steering Committee
TURP: Transurethral resection of prostate
UI: Urinary incontinence
UK: United Kingdom
USI: Urodynamic stress incontinence (urodynamic diagnosis)

## Footnotes

1 Appropriate criteria for proof beyond reasonable doubt cannot be specified precisely. A difference of at least three standard deviation in the interim analysis of a major endpoint may be needed to justify halting, or modifying, such a study prematurely [17] .
